# Experimental Investigation on Water Adsorption Using Laser Photoacoustic Spectroscopy and Numerical Simulations

**DOI:** 10.3390/ma14195839

**Published:** 2021-10-06

**Authors:** Cristina Popa, Mioara Petrus, Ana Maria Bratu, Irina Negut

**Affiliations:** Laser Department, NILPRP-National Institute for Laser, Plasma and Radiation Physics, 409 Atomistilor St., P.O. Box MG-36, 077125 Magurele, Romania; mioara.petrus@inflpr.ro (M.P.); ana.magureanu@inflpr.ro (A.M.B.); negut.irina@inflpr.ro (I.N.)

**Keywords:** silica gel, photoacoustic spectroscopy, numerical simulations, optical technique

## Abstract

In the present research we propose a model to assess the water vapors adsorption capacity of a SiO_2_ trap in the breathing circuit, aiming to reduce the loading of interfering compounds in human breath samples. In this study we used photoacoustic spectroscopy to analyze the SiO_2_ adsorption of interfering compounds from human breath and numerical simulations to study the flow of expired breath gas through porous media. As a result, the highest adsorption rate was achieved with a flow rate of 300 sccm, while the lowest rate was achieved with a flow rate of 600 sccm. In the procedure of H_2_O removal from the human breath air samples, we determined a quantity of 213 cm^3^ SiO_2_ pearls to be used for a 750 mL sampling bag, in order to keep the detection of ethylene free of H_2_O interference. The data from this study encourages the premise that the SiO_2_ trap is efficient in the reduction of interfering compounds (like water vapors) from the human breath.

## 1. Introduction

Human breath is some of the most excellent biofluids in the gas phase that can be simply and noninvasively provided by a single human. It can be used for monitoring human’s metabolic condition [1]. Collecting and analyzing human breath samples is preferred to direct quantification of blood samples due to the fact that it is noninvasive, inexpensive and does not cause physical pain. Moreover, sample contamination is easily avoided [2,3,4]. The inhaled human breath air is an interference of gases, including nitrogen, oxygen, carbon dioxide (CO_2_), and water. There are also other gases present in the inhaled human breath air, like inactive gases and traces of volatile organic compounds (VOCs) but their quantities are very small. The nitrogen content of inhaled air is about 78% by volume and the oxygen in air: oxygen is about 21% by volume, whereas the inhaled CO_2_ is about 0.03–0.04% by volume in air. The percentage of inhaled water vapors in air varies from 0.01 to 4.24% [4,5,6,7,8]. When we breath, we take oxygen from the air and release CO_2_ and water vapors into the air. In the exhaled breath air there is less oxygen, more CO_2_ (around 4% in the exhaled air), and more water vapors (about 5–6%) than in the inhaled breathed air. The amount of nitrogen in the inhaled and exhaled breathed air remains the same. The complex gases in human respiration fluctuate extensively from subject to subject, both qualitatively and quantitatively, in special for VOCs. More than 1000 trace VOCs have been selected in human respiration, at amounts that range from ppmV (parts per million by volume) to pptV (parts per trillion by volume) levels [7,8]. In the case of exhaled breath, VOCs are typical to every person, and include isoprene, acetone, ethane, and methanol, which are the results of core metabolic steps.

In recent years, several new methods and devices have been designed for detecting the presence of a particular substance (e.g., dangerous gas in the air or a trace biomarker of the human breath) [1,2,3,4,5,6,7,8,9,10]. Many spectroscopic instruments, such as Faraday laser magnetic resonance spectroscopy or photoacoustic spectroscopy, have already been used for appreciating a complete area of chemical compounds. All spectroscopic devices can qualitative and quantitative detect the effect of light absorption in a substance. The detectors should be equipped with an efficient sampling system that can eliminate/retain the humidity (water vapors); this issue is not as much debated in the scientific literature. To satisfy the requirement of water interference, miniaturized traps were developed and loaded with Silica gel (SiO_2_) granules/pearls for human breath assessment. A key purpose of a SiO_2_ trap is to retain the moisture in the human respiration without changing the substance concentrations. This is highly important as water vapors interfere with the gases to be analyzed from the human respiration.

Desiccants absorb water and can bind it chemically (reversibly or irreversibly) or physically. The desiccants can be divided into four main categories: non-renewable chemical desiccants, renewable chemical desiccants, silica gels, and molecular sieves. They are suitable for virtually drying all gases and solids. Silica gels have a broad spectrum of applications in the laboratory and in the technical and industrial sectors; possible applications in the laboratory are in desiccators, drying towers, and absorption tubes [8,11,12,13,14,15,16,17].

Over the last years, the photoacoustic spectroscopy laser-based tool was taken into consideration as a high sensibility method, with consideration to the conventional spectroscopic techniques [10,11,12,13,14]. Infrared absorption spectroscopy is an usual technique to acquire spectral signatures of chemical compounds with high resolution and high sensitivity [8,10,17].

In our previous published paper [8], we investigated the problem of interfering CO_2_ from human respiration, by modifying the laser photoacoustic spectroscopy to fit this type of experiment. We analyzed a KOH trap to the measurement system, suitable for CO_2_ removal from the human breath. As a continuation of our previous work, the present study introduces a new scientific procedure for the investigation of a SiO_2_ trap capacity in the efficient reduction of interfering compounds from human breath, in this case, water vapors. The experiments were conducted at the National Institute for Laser Plasma and Radiation Physics, Bucharest, Romania (http://llasem.inflpr.ro, accessed on 29 September 2021). The photoacoustic spectroscopy and numerical simulations were applied to establish the capacity of traps in connection of several verifiable characteristics, such as the size and geometry of the packing.

Using a different method for VOC_S_ detection, other previous research [18] present information about silica gel study for the case of breath acetone analysis as a way for blood glucose monitoring. In the present research, SiO_2_ traps with two nonidentical volumes were tested before and after the interaction with human breath respiration, and the experimental data confirmed the numerical simulation and photoacoustic spectroscopy determinations.At the same time, our work gives a “new look” on breath-based metabolomics by introducing a dedicated optical device taking gas phase by vibrational spectroscopy together with a CFD module [19] to determine the adsorption capacity and humidity with a SiO_2_ trap. As a result, we established the volume and flow rate to be used in order to productively decrease the H_2_O from the breath.

The originality of our research is given by the assessment of healthy participant’s respiration using a modified photoacoustic spectroscopy system together with a numerical simulation. Taking into account the fact that the water vapors present in the human breath interfere with other gases to be analyzed, we updated our photoacoustic spectroscopy system by introducing a new trap with SiO_2_ pearls. The purpose of this trap is to remove the interfering water vapors from human breath samples.

## 2. Materials and Methods

In this scientific investigation, the applicability of photoacoustic spectroscopy together with numerical simulations in the analysis of a person’s respiration before and after the interaction with SiO_2_ orange pearls was determined. We examined the efficiency of traps filled with SiO_2_ pearls and distinct volumes (13 and 213 cm^3^) in eliminating H_2_O from the exhaled human breath air. We determined what model to be used to productively decrease the quantity of the H_2_O from the exhaled human air sample. In particular, we evaluated the C_2_H_4_ in the presence of SiO_2_ orange pearls using the CO_2_ laser photoacoustic spectroscopy.

**Figure 1:**
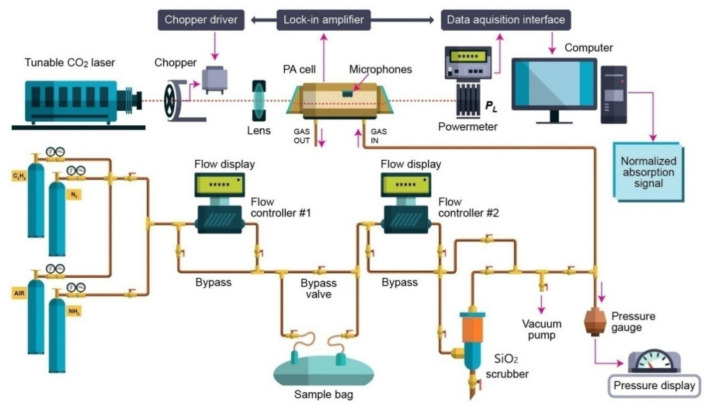
The photoacoustic system for the study of the SiO_2_ adsorption from human respiration.

Our investigation involved the assessment of ethylene (C_2_H_4_) in the presence of SiO_2_ orange pearls by using the CO_2_ laser photoacoustic spectroscopy. The laser photoacoustic system is graphically presented in Figure 1 and discussed in our previous works [8,10,12,13,17,20].

C_2_H_4_ human breath samples were measured before and after passing through the SiO_2_ trap by using photoacoustic spectroscopy to examine the wide applicability of this breath conditioning approach.The CO_2_ laser spectral outputs take place in the wavelength domain where an extended number of molecules perform powerful absorption characteristics and absorptive interferences from water vapors.

CO_2_, and other important atmospheric elements may affect the capacity of determinations. Due to the exact agreement of the H_2_O vibrational rotational transitions with the CO_2_ laser lines, the H_2_O at high concentration in analogy with trace gases (like C_2_H_4)_ is unavoidably excited by CO_2_ laser radiation and the correlated photoacoustic signal can exceed the trace signal by many orders of magnitude. The absorption coefficient increases strongly with the temperature, but it is independent of the H_2_O concentration over a wide range. Given thatthe CO_2_ laser is always tuned at one absorption peak of the CO_2_, this is also an interfering gas in breath analysis. We addressed this issue in our previously published work [8], in which we presented the retention of CO_2_ by introducing a KOH trap in the system measurement.

The gas from the sample bag was sent into the PA (flow rate of 300, 400 and 600 sccm), in order to provide an adequate time flow in the trap column and to reduce any tendency for the vapor to stick to cell walls or any other repercussions of internal out gassing of impurities, which would result in an increase of background signals throughout the experiment.

The photoacoustic detection system is composed from a resonant photoacoustic detector cell (including thevolunteer respiration sample) and a tunable continuously wave CO_2_ laser source. The input power of the laser beam was measured with help of a laser radiometer with a measuring head and the optimum laser power is finally adjusted using the screws supporting the optical resonator components.After the input laser power was measured, the light beam of the laser was modulated with a high quality, low vibration noise, and variable speed 4–4000 Hz by a mechanical chopper model DigiRad C-980, Terahertz Technologies Inc., New York, NY, U.S.A., with 30 slot apertures operated at the appropriate resonant frequency of the PA cell (564 Hz).At the point of insertion of the chopper blade, the laser beam diameter is typically 5 mm and is nearly equal to the width of the chopper aperture. An approximately square waveform was produced with a modulation depth of 100% and a duty cycle of 50% so that the average power measured by the radiometer after the PA cell is half the CW value. By enclosing the chopper wheel in housing with a small hole of 10 mm allowing the laser beam to pass, chopper induced sound vibrations in the air that can be transmitted to the microphone detector.

The sensing path is ensured by a gas mixture scheme designed for the control of gas molecules in the experiments. To measure a complex mixture of gases, it is essential to customize the resonant cavity with a commercially gas mixture and to indicate linear responses of the detector for low detection of a gas. To calibrate the measurement, it is necessary to know the gas absorption coefficient at a given laser wavelength and the cell responsivity. The cell responsivity was experimentally determined by using a reference gas mixture (C_2_H_4_ in nitrogen).

The continuous wave CO_2_ laser is line-tunable, frequency stabilized and produces an emission of energy in the (9.2–10.8) µm domain on 54 various vibrational-rotational lines, with powers fluctuating between (0.5–6.5) W. The CO_2_ laser beam is intensity modulated by a mechanical chopper which works at a suitable resonant frequency of the cell (564 Hz). The laser is focused by a ZnSe lens, and then enters into the photoacoustic cell which is secured with four microphones (where the acoustic wave is detected and produces an equivalent signal). In the experiments, there were four Knowles electret EK-3033 miniature microphones connected in series and mounted flush with the wall. Each microphone has a sensitivity of 20 mV/Pa and a total sensitivity of 80 mV/Pa. They are positioned at the loops of the standing wave pattern, at an angle of 90° to one another. The microphones are fixed to the resonator by holes with a 1 mm diameter, positioned on the central perimeter of the resonator. The battery-powered microphones are mounted in a Teflon ring pulled over the resonator tube. The signal is fed into a lock-in amplifier that provides the amplitude and phase of the photoacoustic signal. The value of the acoustic signal determined by microphones and normalized to the size of the CO_2_ laser radiation power is comparable to the molecular absorption coefficient of the analyzed gas sample at a CO_2−_ used laser radiation wavelength. A power-meter measures the laser beam power after the photoacoustic cell and its digital output is collected by the data acquisition interface module in the output from the lock-in amplifier. All the experimental results are transformed and stored in a computer [8,10,20]. The gas handling system is a significant section of the experimental set-up for the gas determinations. This system assures the gas purity in the photoacoustic cavity. Furthermore, it can be used to pump out the cavity and to fixup the breath sample in the cavity, keeping the total and partial pressures of gas mixtures. The gas handling system comprises two gas flow controllers: the MKS 1179A (0–1000 sccm) [sccm-standard cubic centimeters per minute, 1 sccm at 0 °C = 7.436 × 10^−7^ mol/sec] and MKS 2259CC (0–200 sccm), which are in contact with a digital MKS 247C four-channel apparatus [8,20,21].

In the CO_2_ photoacoustic spectroscopy method, the resulting signal, processed by the sensitive detector, is directly proportional to the absorption coefficient and the laser power. First, we analyzed the experimental values of C_2_H_4_ absorption coefficients for all laser wavelengths (called signatures), unique for the laser frequency and C_2_H_4_ molecules. These signatures are absolute entities and provide the specifics of instrument performance in terms of detection limit and interference rejection.

The breath has been analyzed for a single subject/10 samples/day for a period of 3 months. The volunteer was non- or ex-smoker, non-alcoholic, non-renal, non-diabetic without chronic mental or physical health problems, and without any recent antibiotic therapy. Prior to the analysis of breath, the volunteer was asked to avoid for at least 6 h before or at any time during the breath sample collection: alcohol and coffee, food or beverages, and to refrain from exercise in the morning. On the day prior to the test, products such as onions, leeks, eggs, and garlic should be avoided. Information was asked regarding age, body weight and body height, time and nature of the least meal and drink, recent exercise activity, medication, and smoking status.

**Figure 2:**
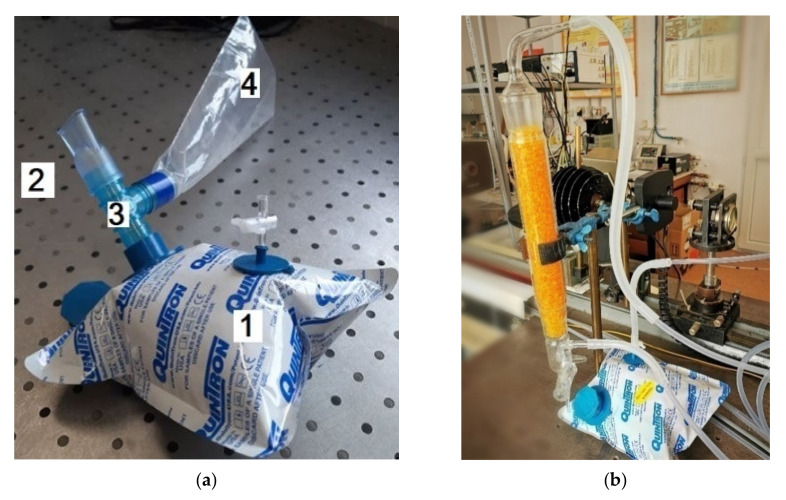
(**a**): 1—0.75 L Aluminium-coated bags (QuinTron, USA); 2—Mouthpiece; 3—Tee connector; 4—0.40 L Discard bag;(**b**) Connected SiO_2_ trap and aluminum-coated bags.

All the respiration samples were collected in 0.75-L bags coated with aluminum and designed to accumulate and keep (for a maximum of 6 h) multiple respirations [10]. The volunteer positioned the piece in theirmouth (Figure 2a), forming a fixed seal around it with its lips, and then naturally exhales through the mouth. When a proper respiration is collected, the volunteer stops the natural exhalation. The bags with the collected breath are delivered to the laboratory and transferred into the measuring cell (where we can detect the traces of gases by the gas flow controller #2 (MKS 1179A). Before entering the photoacoustic cell, the gas mixture moves in a specified direction via a SiO_2_ trap (Figure 2b), which holds most of the interfering water vapors. The elimination of H_2_O is limited to the absorbent surface of the pearls so, the higher the surface area, the larger the ability of the trap system to absorb the water molecules.

In our experiments, we used SiO_2_ orange typically Roth pearls of (2–5) mm(Figure 2b), with the following properties: total adsorption capacity similar to blue gel (Silica Gel Blue), large surface (approx. 750 m^2^/g)which enables high absorption capacity of steam, adsorption capacity: approx. 40 wt% at 80% relative humidity, patented humidity indicator—heavy metal-free, color change from orange to colorless at approx. 6 wt% load, drying pearls orange: boiling point (bp) >999 °C, melting point (mp) >550 °C, regeneration at 130 °C, ≈ 4 h.

The transfer of the sample gas from the aluminum bag to the cell was achieved at a controlled flow rate of 300, 400, or 600 sccm, and the pressure of gases entered in the cell was established with a Baratron pressure gauge. This way, the time required for the sample gas to pass through the SiO_2_ trap is approximately ~1.25 min for a flow rate of 600 sccm, ~1.87 min for a flow rate of 400 sccm and ~2.5 min for a flow rate of 300 sccm.The final pressure inside the cell, measured for breath samples from healthy humans, is usually at ~800 mbar (this pressure is the result of the initial pressure in the sample bag and the bag and cell volumes, respectively). In addition to photoacoustic spectroscopy determinations, we built a CFD module while the SiO_2_ adsorption process is simulated at room temperature for the exhaled breath experiments. We study the changes of adsorption, temperature, pressure, and initial water vapors concentration during the adsorption processes. To simulate the adsorption of water vapor by SiO_2_ pearls with a CFD model, we used Comsol by Laminar Flow, Heat and Moisture Transfer [19] with an interface that computed the velocity and adsorption capacity for the flow of a single-phase fluid in the laminar flow regime [21].

A special attention is required for the adsorption system, particularly the performance of the adsorbent bed, which is one of the most important parts in the system andresearch on CFD simulation on adsorbent bed in adsorption system for exhaled human breath (as moist air) is still rare. The SiO_2_ water adsorption process can be described as the contour of the temperature, pressure, and adsorption. The simulation can be described by using the 2D geometry model or the 3Dgeometry model within the porosity model. In the present study, we used the simple 3D model to get more detail about the process. In the 3D Geometry of SiO_2_ packing, absorption of SiO_2_ packaging and space between beds will depend on the size of SiO_2_. Therefore, we designed a spherical SiO_2_ pearl with a diameter of 3 mm and then cloned it 128 times in order to obtain a SiO_2_ packed-bed. The radii of SiO_2_ pearls were reduced by approximately 2% and the simulation was performed in a tetrahedral mesh. Therefore, we used a cylinder with a radius of 8.2 mm and a height of 30 mm. In this cylinder we have 128 spheres of SiO_2_, each with a diameter of 3 mm. This geometric model was limited by the computation unit. For the simulation we have used a XPS 15 7590 with I9-9980HK CPU @ 2.4 GHz and 32 GB RAM.

The energy consumption and speed of analysis was the same for all the parameters used once the physical model and the mash was the same.

In addition to numerical simulations and photoacoustic spectroscopy determinations, an analytical microbalance was used for the weighting SiO_2_ pearls from the trap. For the weight measurements a Partner Radwag Mya 0.8/3.3Y analytical microbalance with an accuracy of ±3 μg was used. In order to highlight water absorption from human breath by SiO_2_ trap, we randomly selected five pearls to be weighted before and after experiments. Before experiments, the total mass of the pearls was of 0.247050 g. As expected, after the experiment, the mass of the used pearls increased with 2.81%. Another indicator of water absorption is the color difference between the pearls before and after the experiments.

## 3. Results

### 3.1. CO_2_ Photoacoustic Spectroscopy Application in the SiO_2_ Traps Assessment

**Figure 3:**
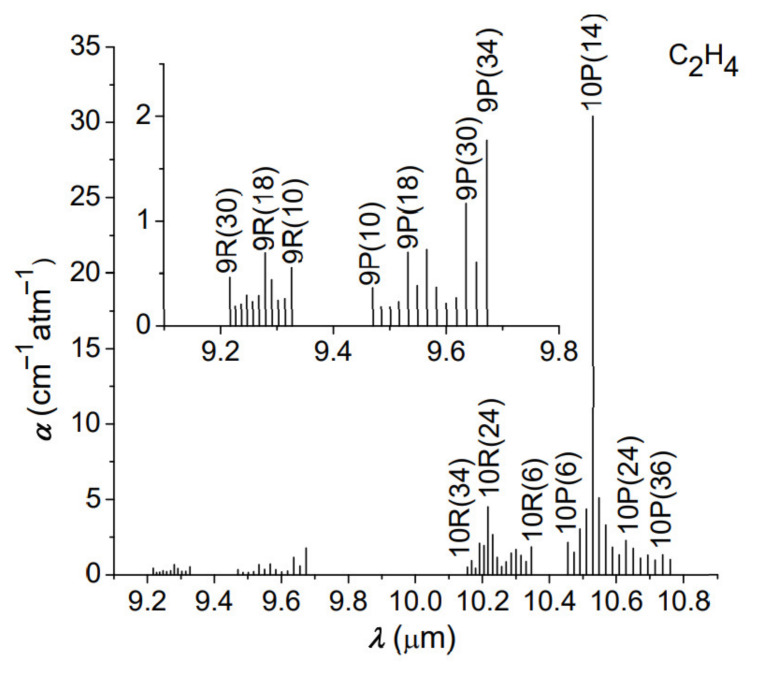
C_2_H_4_ absorption coefficients for all laser wavelengths.

As one can observe from Figure 3, the corresponding C_2_H_4_ absorption concentration at every laser wavelength was determined and the maximum absorption for C_2_H_4_ was at 10.53 µm, which corresponds to 10P(14) CO_2_ laser line. The image below shows an enlarged view for the 9-μm band determinations.

**Figure 4:**
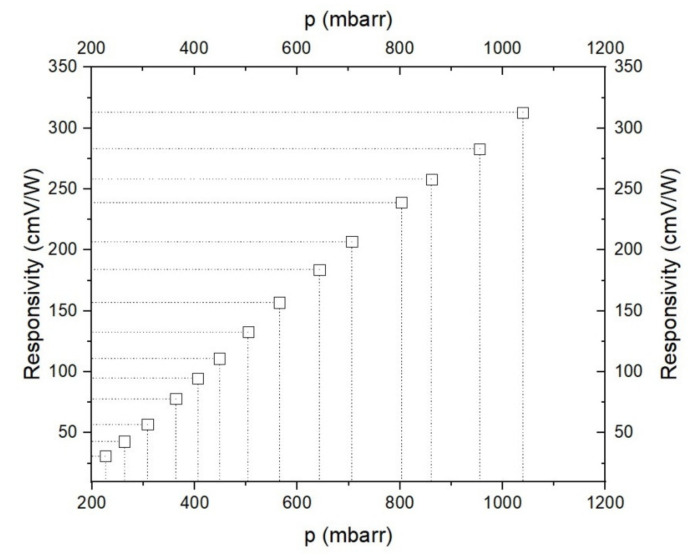
The responsivity of the photoacoustic cell.

An important parameter in the measurements is represented by responsivity R (cmV/W) of the cell, which depends on the gas pressure inside the cell (Figure 4).

Taking into account the fact that the initial pressure in the sample bags filled by the volunteers differs from one case to another, it is important to know the pressure dependence of the cell responsivity (Figure 4).

The responsivity of the cell was determined by using a calibrated mixture (Linde Gas) of 0.96 ppmV (2%) C_2_H_4_ diluted in nitrogen 6.0 (purity 99.9999%). The pressure dependence of the responsivity was always measured at the center of the CO_2_ laser line by using a frequency stabilized laser (instability 3 × 10^−l^).

The air samples were collected from the same subject repeatedly (healthy volunteer, 37 years old) and with a new filling of SiO_2_ pearls. The respiration from the sample bag was sent into the PA cell at a directed flow rate of 300, 400 and 600 sccm. The resulting pressure within the PA cell was around 800 mbar and the equivalent responsivity was 240 cmV/W.

**Figure 5:**
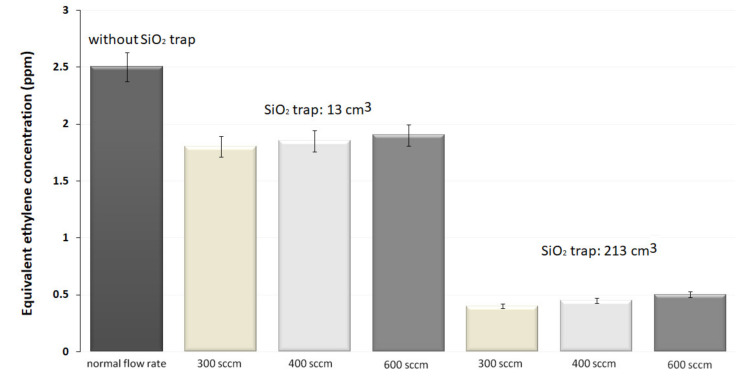
Investigation of SiO_2_ traps for H_2_O removal from human respiration.

The results recorded in the absence of the SiO_2_ trap presented a corresponding C_2_H_4_ absorption concentration of 2.5 ppmV, showing mostly the contribution of C_2_H_4_, H_2_O vapors, and CO_2_ to the absorption of 10P(14) CO_2_ laser line (Figure 5). In the case of 13 cm^3^ trap of SiO_2_ pearls, we registered a decrease of the photoacoustic signal with an equivalent C_2_H_4_ concentration of about 1.8, 1.85 and 1.9 ppm for 300, 400 and 600 sccm, respectively. For the trap with the volume of 213 cm^3^, the measured C_2_H_4_ concentrations were of 0.4, 0.45, and 0.5 ppm.

**Figure 6:**
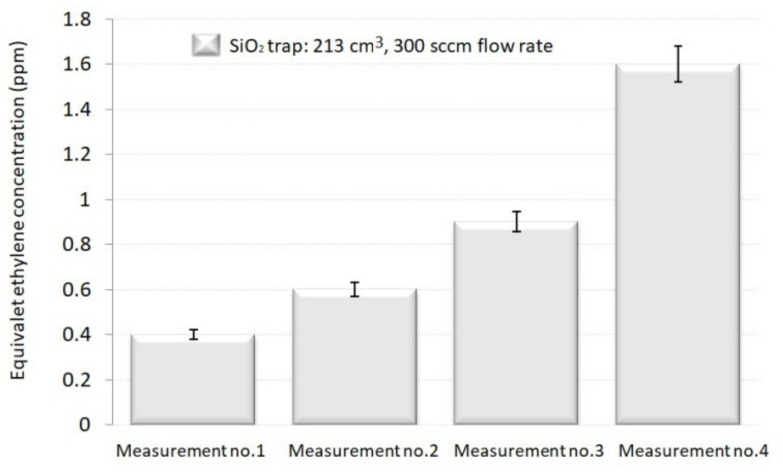
Decrease of SiO_2_ traps capacity when the identical replenish was used for multiple tests.

In the case of the larger SiO_2_ trap (volume of 213 cm^3^ and a controlled flow rate of 300 (sccm) in the H_2_O removal (Figure 6), the equivalent C_2_H_4_ concentration increased by 1.5. times for the second run, by 2.25 times for the third, and by 4 times for the fourth.

### 3.2. Numerical Simulation in the SiO_2_ Traps Evaluation

We studied the changes and contour of the temperature and pressure from the exhaled breath bag, and the adsorption during the adsorption processes. Table 1 presents the values used in the 3D model simulation.

**Figure 7:**
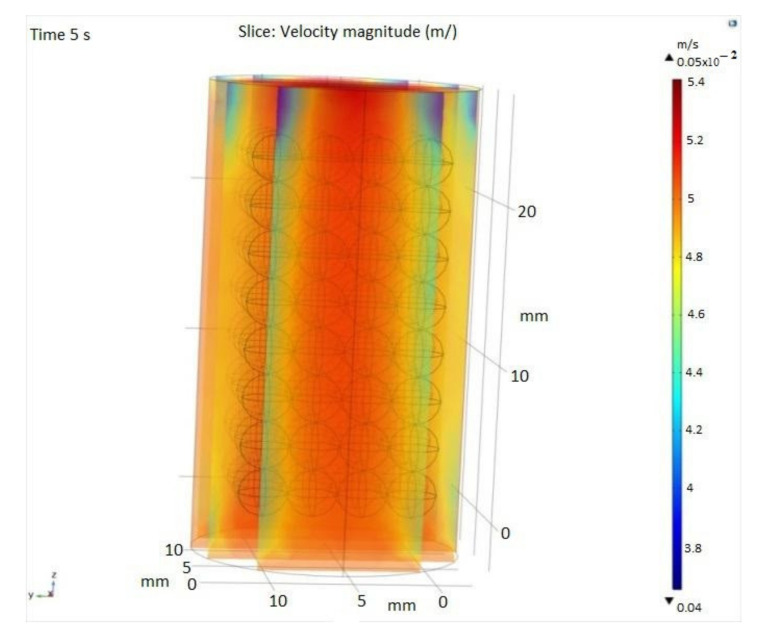
3D velocity profile expressed in m/s.

Figure 7 illustrates the relative humidity in the SiO_2_ packed bed and the water vapors trajectory in the SiO_2_ bed along the *Z* axis and over a period of 5 s.

**Figure 8:**
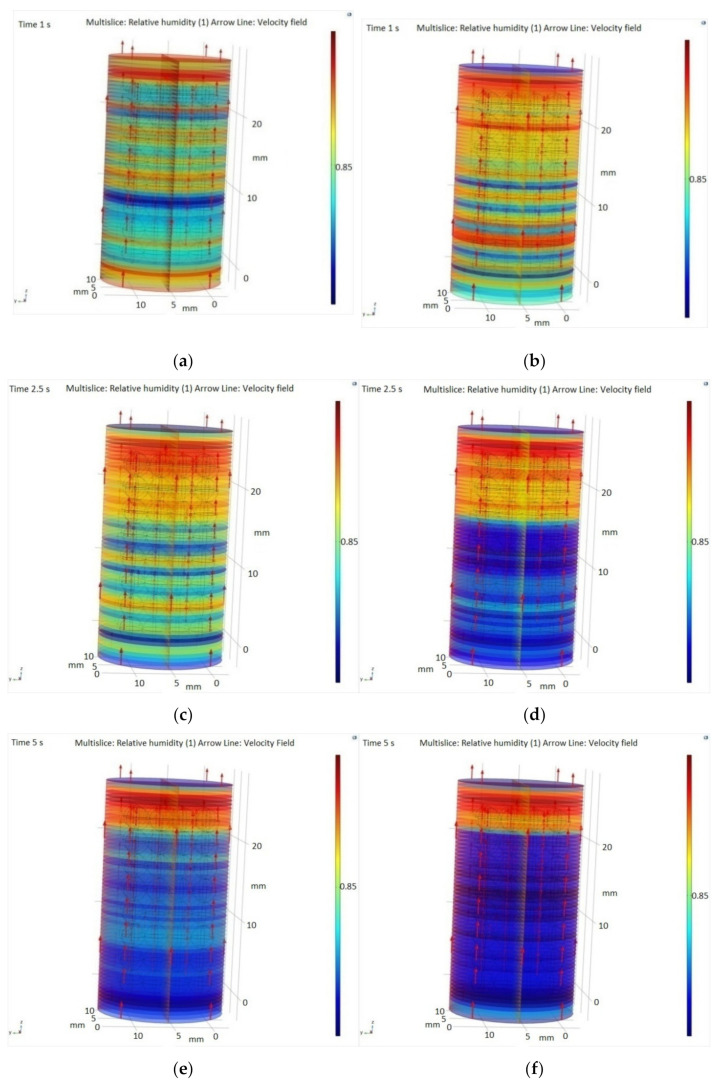
3D concentration profile of the relative humidity and water vapors molecules trajectory in the SiO_2_ bed along the *z* axis, at different time moments (t = 1, t = 2.5, and t = 5 s) and at a flow rate of 300 (**a**,**c**,**e**) and 600 sccm (**b**,**d**,**f**).

Figure 8 shows the water adsorption process capacity of the SiO_2_ packed bed at two different flows rates. At a flow rate of 300 sccm, the humidity evolution in time (after 1, 2.5 and 5 s) is presented in Figure 8a–c and at 600 sccm in Figure 8d–f. These numerical simulations aimed to evaluate the adsorption capacity of SiO_2_ pearls in order to determine the optimal input flow of the gas sample.

As a result, during the adsorption process, the SiO_2_ adsorption distribution shows that the adsorption in the central area of the granules is higher, while the SiO_2_ layers increase in the whole volume when using a flow of 300 sccm. In comparison, at a flow speed of 600 sccm, the adsorption increases only in the outer layer. Therefore, the highest adsorption rate is achieved at a flow rate of 300 sccm, while the lowest rate is obtained at 600 sccm.

**Figure 9:**
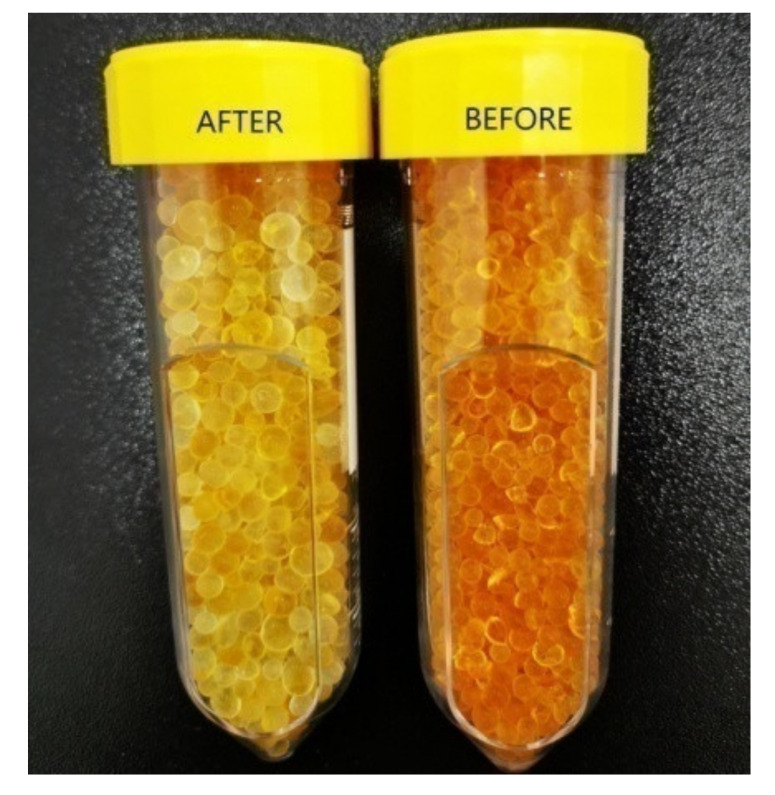
Picture representing the SiO_2_ orange pearls after the interaction with a breath sample and before the interaction with a breath sample (color change from orange—before: right to colorless—after: left).

Another indicator of water absorption is the color difference between the pearls before and after the experiments (Figure 9).

## 4. Discussion

The current research assessed the influence of SiO_2_ on water vapor adsorption in the respiration route.

The results recorded in the absence of the SiO_2_ trap presented a corresponding C_2_H_4_absorption concentration of 2.5 ppmV, showing mostly the contribution of C_2_H_4_, H_2_O vapors, and CO_2_ to the absorption of 10P(14) CO_2_ laser line.

Initially, we have used the 13 cm^3^ trap of SiO_2_ pearls, and we registered a decrease of the photoacoustic signal with an equivalent C_2_H_4_ concentration of about 1.8, 1.85and 1.9 ppm for 300, 400 and 600 sccm, respectively. This indicates that the H_2_O concentration was reduced by factors of 1.4, 1.35 and 1.3, as compared with previous measurements. A greater size of the SiO_2_ trap demonstrated to be more operative for eliminating H_2_O from the exhaled air. For the trap with the volume of 213 cm^3^, the measured C_2_H_4_ concentrations were of 0.4, 0.45 and 0.5 ppm, showing that the H_2_O concentration was attenuated by factors of 6.25, 5.55 and 5, as compared with previous measurements. By using larger traps, a higher transfer rate of the gas mixture in the photoacoustic cell is manageable; by enlarging the flow rate to 600 sccm. With the largest volume, we diminished the H_2_O content from the exhaled air at a level at which is no longer influencing the C_2_H_4_, a fact proved by the constant evolution in time of all parameters. Consequently, the trap is effective for only a large quantity of SiO_2_ pearls.

In addition, we determined the efficiency of the SiO_2_ trap that demonstrated to be more operative (volume of 213 cm^3^ and a controlled flow rate of 300 sccm) in the H_2_O removal. An obvious saturation result was seen: the SiO_2_ trap was no longer efficient when the same replenish is used for several runs and cannot fully absorb the H_2_O from the gas mixture. The equivalent C_2_H_4_ concentration increased by 1.5. times for the second run, by 2.25 times for the third, and by 4 times for the fourth one. It was determined that a new replenish of SiO_2_ trap should be inserted after each assessment.

An interesting finding can be found in an article conducted by Gregory E. Cmarik et al. [22]. By using a different investigation method this study presents the analysis of performance degradation of SiO_2_ after extended use onboard the ISS. Water vapor adsorption tests were conducted on vials of desiccant which was sampled from the first sorbent layer exposed to the incoming air. The results clearly show roughly an 85% loss in performance. In a study by Li C. et al. [23] it was proved that the area of silica holds silanol or siloxane groups that allow a distinctive interaction with water (hydrophilic), representing a particular polar group having similar properties with water. Practically, the hydrogen bonds were formed by the reaction of water and the hydroxyl group [23,24,25], and it is rational to think that water vapor adsorption, depending on the amount of the hydroxyl group on the silica surface and the adsorption ability, becomes greater in size with the hydroxyl content. By investigating a different adsorption compound with different investigation method Song et al., [25] concluded that SiO_2_ is an adsorbent and a considerable number of investigations have been conducted to find specific properties of SiO_2_ pearls [22,23,24,25], but none with the help of the photoacoustic technique.

A special attention in this manuscript was paid to numerical simulations building a CFD model used to simulate the adsorption of water vapor from the exhaled breath onto silica gel porous media. To get more details about the absorption phenomena, we used the 3D Geometry of SiO_2_ packing, where the absorption of SiO_2_ packaging and space between beds depends on the size of SiO_2_.

The results showed that a high concentration of water vapors was retained by the porous material represented by SiO_2_, and the humidity increases inside the pearls. It was observed that the highest adsorption rate is achieved with a flow rate of 300 sccm.

In addition to numerical simulations and CO_2_ photoacoustic spectroscopy determinations, an analytical microbalance was used for the investigation of the SiO_2_ trap. As expected, from weight measurements, the mass of the used pearls increased by 2.81%. The purpose of our research consists in finding new solutions for the phenomena of water vapors retention from the human breath respiration, giving new tools for maintaining of a healthy human respiration.

Supplementary opportunities can be enlarged for the persons who carried out scientific research to increase new and improved ways for the investigation of the SiO_2_ trap capacity in the efficient reduction of interfering compounds from human breath, like water vapors, using gas sensing determinations and numerical simulations.

## 5. Conclusions

In summary, the current work was carried out by implementing a methodology that assured better conditions to measure real concentrations of gases from the exhaled human breath. In the procedure of H_2_O retention from the human respiration samples, we experimentally obtained, a quantity of 213 cm^3^ SiO_2_ pearls to be used for a sampling bag of 750 mL in order to keep the detection of C_2_H_4_ free of H_2_O interference. It should be mentioned that this volume of 213 cm^3^ must be re-evaluated for samples with a larger volume or in conditions of increasing the gas flow rate. Furthermore, a CFD model was built and used to study the water vapors’ removal from the exhaled breath. The simulation results showed that a high concentration of water vapors is retained by the porous material represented by SiO_2_, and the highest adsorption rate was achieved with a flow rate of 300 sccm, when we compared it with a speed of 400 and 600 sccm. As a general conclusion, the results from this research maintain the premise that the SiO_2_ trap is efficient in the reduction of interfering compounds from human breath, like water vapors. Furthermore, the results from the present research showed that a high concentration of water vapors was retained by the porous material represented by SiO_2_, and the humidity increases inside the pearls.

## Figures and Tables

**Table 1:** Parameters used in the 3D model simulation.

Parameters	Value
Temperature [K]	298.15
Pressure [mbarr]	800
Fluid velocity [m/s]	0.5
cH_2_O [ppm]	25
The radius of the silica gel sphere [mm]	15
Density (kg/m^3^) [20]	730
Porosity [20]	0.36
Specific heat capacity (J/kg∙K) [20]	921
Thermal conductivity (W/m∙K) [20]	0.174

## Data Availability

Not applicable.
